# Molecular Characterization of a Tolerant Saline-Alkali *Chlorella* Phosphatidate Phosphatase That Confers NaCl and Sorbitol Tolerance

**DOI:** 10.3389/fmicb.2021.738282

**Published:** 2021-09-28

**Authors:** Jingang Wang, Qinghua Shan, Ye Ran, Dexiang Sun, Haizhen Zhang, Jinzhu Zhang, Shufang Gong, Aimin Zhou, Kun Qiao

**Affiliations:** College of Horticulture and Landscape Architecture, Northeast Agricultural University, Harbin, China

**Keywords:** tolerant saline-alkali *Chlorella*, NaCl, sorbitol, subcellular localization, gene expression

## Abstract

The gene encoding a putative *phosphatidate phosphatase* (*PAP*) from tolerant saline-alkali (TSA) *Chlorella*, *ChPAP*, was identified from a yeast cDNA library constructed from TSA *Chlorella* after a NaCl treatment. *ChPAP* expressed in yeast enhanced its tolerance to NaCl and sorbitol. The ChPAP protein from a GFP-tagged construct localized to the plasma membrane and the lumen of vacuoles. The relative transcript levels of *ChPAP* in *Chlorella* cells were strongly induced by NaCl and sorbitol as assessed by northern blot analyses. Thus, ChPAP may play important roles in promoting Na-ion movement into the cell and maintaining the cytoplasmic ion balance. In addition, ChPAP may catalyze diacylglycerol pyrophosphate to phosphatidate in vacuoles.

## Introduction

Phosphatidate phosphatase (PAP) has the effects of catalyzing the dephosphorylation of phosphatidate (PA), and generating diacylglycerol and inorganic phosphate (Smith et al., 1957). It is also an essential enzyme in lipid metabolism, plays important roles in lipid synthesis, and is involved in the generation or degradation of lipid signaling molecules (Brindley and Pilquil, 1984; Carman, 1997; Nanjundan and Possmayer, 2001). The PAP enzymes are divided into Mg^2+^-dependent PAP1 or Mg^2+^-independent PAP2 [also be called lipid phosphate phosphatase (LPP) or diacylglycerol pyrophosphate (DGPP) phosphatase] based on the cofactor requirement for catalytic activity (Jamal et al., 1991; Brindley et al., 2002). The PAP1 enzymes play roles in cell homeostasis and lipid synthesis (Han et al., 2006; Sherr et al., 2017; Hassaninasab et al., 2019), and PAP1 enzyme, PAH1, performs catalytic function to regulate phospholipid synthesis on the nuclear and endoplasmic reticulum (Eastmond et al., 2010; Hassaninasab et al., 2019). The absence of *Pahp1* (encoded PAH1) leads to the upregulated of *V-ATPase* (Sherr et al., 2017). The expression of *PAH1* is induced in the absence of Zn (Soto-Cardalda et al., 2012). The PAH1 protein of the fungal pathogen *Candida albicans* restricts viral replication by affecting phospholipid synthesis and plays significant roles in hyphal growth and environmental stress regulation (Mu et al., 2019). In this study, the PAP2 enzymes are highlighted. PAP2, encoded by DPP1 and LPP1, is a vacuole membrane-associated enzyme that catalyzes DGPP to form PA and then catalyzes PA to form diacylglycerol by removing the phosphate (Wu et al., 1996; Han et al., 2001). PAP2 enzymes contain a three-domain lipid phosphatase catalytic motif containing the conserved sequences KxxxxxxRP (domain 1), PSGH (domain 2), and SRxxxxxHxxxD (domain 3). The conserved arginine residue in domain 1 and the conserved histidine residues in domains 2 and 3 are essential for the catalytic activities of PAP2 enzymes (Hemrika et al., 1997; Neuwald, 1997; Stukey and Carman, 1997; Toke et al., 1998, 1999a,b; Zhang et al., 2000; Han et al., 2004). The PAP2 enzymes are localized on the hydrophilic surfaces of the membrane (Stukey and Carman, 1997; Toke et al., 1998, 1999a,b; Zhang et al., 2000; Han et al., 2004), the vacuole (Han et al., 2001, 2004), and Golgi (Huh et al., 2003) and have broad substrate specificity levels and may function under stress conditions (Oshiro et al., 2003). In the early studies, PAP2 enzymes are found that are responsible for lipid signaling in yeast and mammals (Carman, 1997; Nanjundan and Possmayer, 2001; Brindley, 2004; Pyne et al., 2005). In plants, *Arabidopsis thaliana AtLPP1* appears to be more highly expressed in the leaves and roots compared with other tissues, and the expression level of *AtLPP1* increased in Arabidopsis after ionization and UV-B irradiation (Brindley, 2004; Pierrugues et al., 2011). In addition, the *AtLPP2* appears to be expressed at similar levels in all the plant’s tissues, and *AtLPP2* is involved with abscisic acid signaling and regulation of stomatal movements (Paradis et al., 2011).

The *PAP2* gene has been also identified in microalgae (eukaryotic microbes), including *Chlorella variabilis* (Blanc et al., 2010), *Chlorella protothecoides* (Gao et al., 2014), *Chlamydomonas reinhardtii* (Deng et al., 2013), and *Coccomyxa subellipsoidea* (Blanc et al., 2010). However, there are limited reports on the cloning and functional analyses of *PAP2* genes of microalgae. Some studies indicated that the expression levels of citrate synthase and phosphoenolpyruvate carboxylase 1 in *Chlamydomonas reinhardtii* are decreased, but the *PAP2* has higher expression in the RNAi transgenic *Chlamydomonas* strains (Deng et al., 2013, 2014). In our previous study, we determined that tolerant saline-alkali (TSA) *Chlorella* can survive in an environment containing 600-mm NaCl, and the TSA *Chlorella PAP* gene was isolated from a TSA *Chlorella* full-length cDNA yeast library constructed under 1-M NaCl-stress conditions (Qiao et al., 2015). Here, we determined the growth rates of transgenic yeast on a solid medium under high salinity and drought conditions. The subcellular localization of the ChPAP protein in yeast cells was detected using confocal microscopy, and the effects of high salinity and drought conditions on *ChPAP* expression were investigated.

## Materials and Methods

### *Chlorella* Source, Culture, and Gene

The TSA *Chlorella* was previously isolated from extreme saline-alkali soil on the Songnen Plain, Heilongjiang Province, China (Wang et al., 2011), which is rich in different salt types, including NaCl and NaHCO_3_, and grown in liquid Bold’s basal medium (BBM, Bold and Wynne, 1984). The culture conditions were 23°C under a 16-h light/8-h dark photoperiod. The illumination intensity was 40-mmol photons m^−1^ s^−1^. The TSA *Chlorella* cells were maintained in solid BBM, and the sub-culturing and rapid propagation of TSA *Chlorella* was cultured in liquid BBM. A full-length cDNA yeast library of TSA *Chlorella* was constructed (Qiao et al., 2018). A sequence screened from the 1-M NaCl-treated TSA *Chlorella* library had close similarity levels to sequences of other species’ *PAP* genes. Accordingly, it was named *ChPAP*.

### Sequence Analysis

The full-length *ChPAP* sequence was analyzed using BlastX and ORFfinder on the NCBI Web site.^1^ GeneDoc 3.0 software was used to align the sequences of the ChPAP protein and other species. The maximum-likelihood-based phylogenetic tree was constructed using MEGA 5.1 software. The transmembrane domains in the ChPAP sequence were predicted using the TMHMM server v. 2.0.^2^

### Plasmid Construction, Yeast Transformation, and Stress-Tolerance Assays

The *ChPAP* cDNA fragment harboring the open reading frame was amplified from TSA *Chlorella* using PCR with the ChPAP-forward (5'-GGATCCATGTTGCACGCGATGGTGG-3'; BamHI restriction site) and -reverse (5'-GTCGACTCAAACAGGCACCATGCTGC-3'; KpnI restriction site) primers and Phanta Max Super-Fidelity DNA Polymerase (Vazyme Biotech Co., Ltd., Nanjing, China). For yeast transformation, *ChPAP* was ligated into the pYES2 vector (Invitrogen, United States) digested with BamHI and NotI restriction enzymes to construct the plasmid pYES2-*ChPAP*, which was transferred into InVSC1 yeast cells using the PEG/LiAC method (Gietz et al., 1992). First, to test the tolerance of transgenic yeast under different stress conditions, yeast cells containing, independently, the pYES2-*ChPAP* and empty pYES2 vectors were cultivated in liquid SD-Uracil (pH 5.8) medium at 30°C for 2days. The concentration of the transgenic yeast cells was adjusted to an OD_600_ value of 0.5 and then diluted to 10^−1^, 10^−2^, 10^−3^, and 10^−4^ with sterile H_2_O. A total of 4.5μl of each dilution series was placed into a solid yeast (1% yeast extract, 2% peptone, and 2% galactose) medium supplemented independently with 0.8-M NaCl, 1.0-M NaCl, and 1.6-M sorbitol. The empty pYES2 vector in yeast cells was used as a control, and the growth of yeast cells at 30°C was observed and photographed for 5–10days.

### Subcellular Localization of the ChPAP Protein in Yeast

To determine the subcellular localization of ChPAP, the expression plasmid pYES2-*ChPAP*-EGFP was constructed. The *ChPAP* full-length sequence with restriction sites was cloned using PCR with EGFP-specific forward GFP-F (5'-GGATCCATGTTGCACGCGATGGTGGAC-3') and reverse GFP-R (5'-GGTACCCCAACAGGCACCATGCTGCTTGC-3') primers. The PCR product was ligated into the pEGFP plasmid (Clontech) digested with the BamH1 and KpnI sites. The *ChPAP*-pEGFP fusion fragment was digested at the BamHI and NotI sites in the pEGFP plasmid to construct the empty pYES2 vector.

The pYES2-*ChPAP*-EGFP and pYES2-EGFP plasmids were transferred independently into yeast cells. The transgenic yeast cells were pre-cultured in liquid medium containing 1% yeast extract, 2% peptone, and 2% glucose at 30°C for 2days. Afterward, they were washed three times to remove the remaining glucose. The EGFP and *ChPAP*-EGFP plasmids in yeast cells were induced to express in liquid yeast (1% yeast extract, 2% peptone, and 2% galactose) medium at 30°C for 6h, and then, *ChPAP*-EGFP yeast cells were incubated at 30°C with 20-μm FM4-64 dye for 3h. The remaining dye was removed by washing three times with sterile H_2_O before samples were observed. The fluorescence was detected using laser-scanning confocal imaging system (Olympus Fluoview, FV500). The EGFP and FM4-64 signals were excited at 488nm and 543nm, respectively.

### Expression Analysis of *ChPAP*

To investigate the *ChPAP* transcript levels under high salinity and drought stresses, the TSA *Chlorella* samples were grown on medium supplemented independently with 200-mm NaCl and 300-mm sorbitol. The samples were collected at 0, 3, 6, 12, 24, and 48h and then ground with a mortar and pestle in liquid nitrogen for RNA isolation.

The total RNA of TSA *Chlorella* was extracted using RNAiso Plus reagent (TaKaRa, Japan). The ChPAP-specific forward (5'-ATGGGCCTCAAGGAAGAC-3') and reverse (5'-TCAAGCGTACTTCGCCTTCAG-3') primers were used to amplify the cDNA probes using a PCR Digoxigenin Probe Synthesis kit (Roche, Switzerland). The northern blot analysis was performed in accordance with a previously published protocol (Qiao et al., 2018).

## Results and Discussion

### Characterization of *ChPAP* Gene

**Figure 1:**
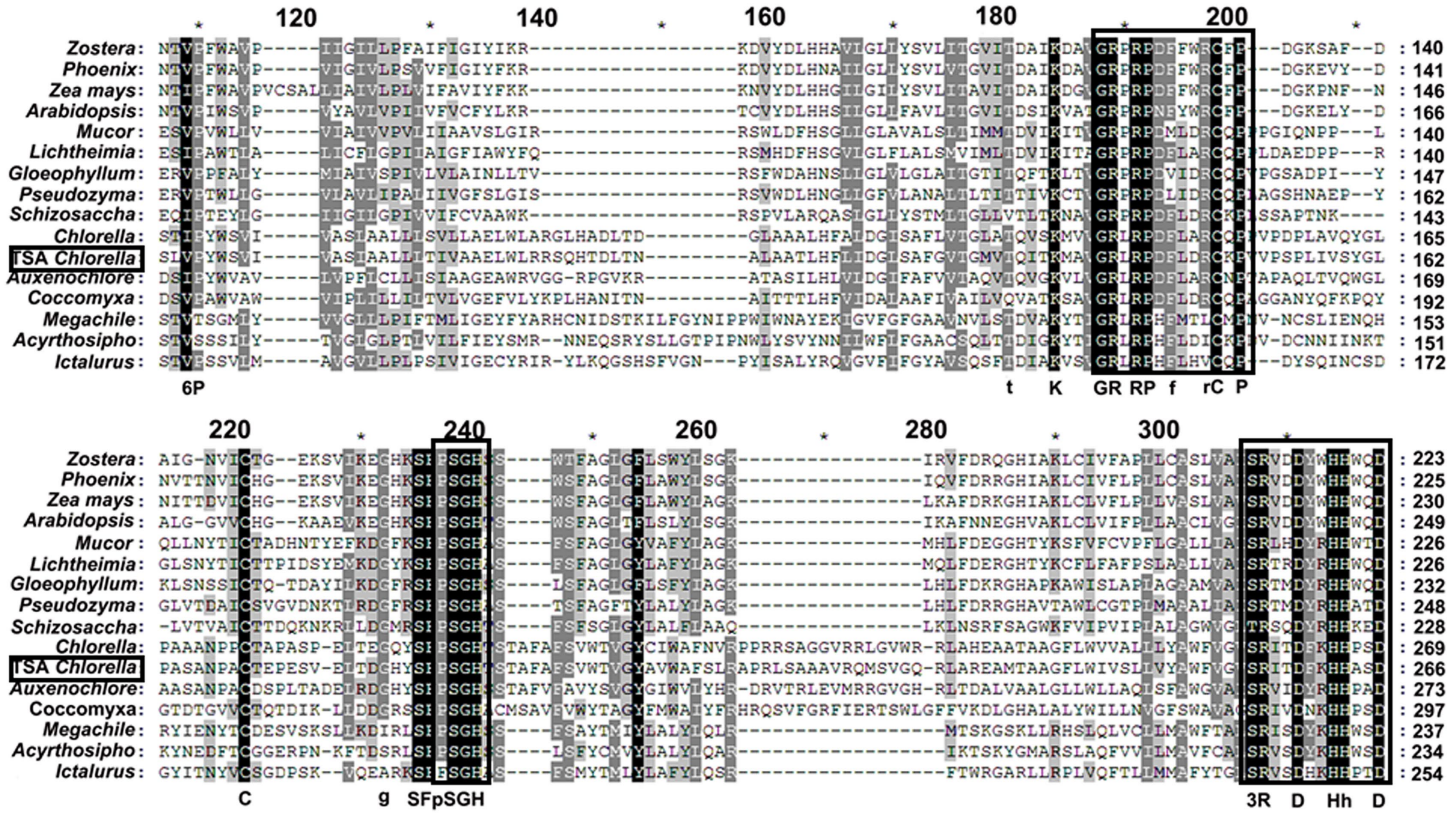
Sequence alignment of the phosphatidate phosphatase (PAP) domains from tolerant saline-alkali (TSA) *Chlorella* ChPAP2 with those of other species. The conserved residues, including the KxxxxxxRP (domain 1), PSGH (domain 2), and SRxxxxxHxxxD (domain 3) motif, are indicated by boldface in boxes. The ChPAP2 sequence data have been deposited in the GenBank database and assigned accession no. KT750011. The other PAP protein sequences were downloaded from GenBank.

**Figure 2:**
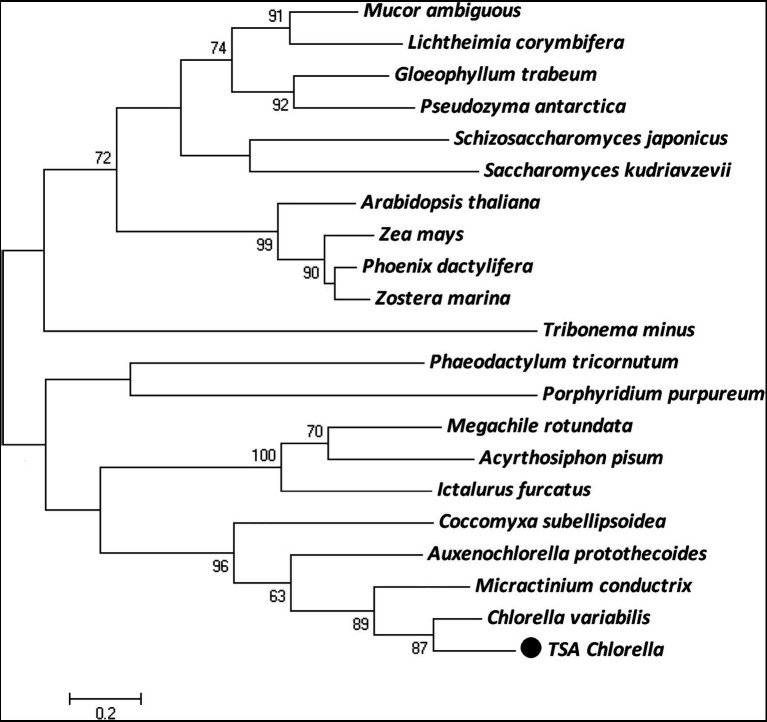
The maximum-likelihood (ML) phylogenetic relationships of PAP proteins between TSA *Chlorella* sp. and those of other species. Bootstrap values were calculated 1,000 times, and values below 50% were not included. The TSA *Chlorella* sp. is indicated by boldface in a box.

The *ChPAP* nucleic acid sequence contained an open reading frame of 1,002 nucleotides that translated into 333 amino acids. The ChPAP amino acid sequence shared close similarities with the previously reported PAP sequences of other species, such as 70% similarity with *C. variabilis* and 53% similarity with *Micractinium conductrix*. A comparison of the ChPAP domains with those in PAPs of other species revealed the presence of conserved domains 1, 2, and 3 (Figure 1), which contained two arginine, one histidine, and two histidine residues, respectively. These results were consistent with previously reported PAP2 protein structures (Hemrika et al., 1997; Neuwald, 1997; Stukey and Carman, 1997). The phylogenetic analysis showed that ChPAP was closely related to the PAP of *C. variabilis* (XP_005848979.1). The TSA *Chlorella* firstly clustered with unicellular microalgae, and then clustered with microbes, animals, and plants (Figure 2). Thus, *ChPAP2* is the *PAP* gene of TSA *Chlorella*.

### Overexpression of *ChPAP2* in Yeast Enhanced Tolerance to NaCl and Sorbitol

**Figure 3:**
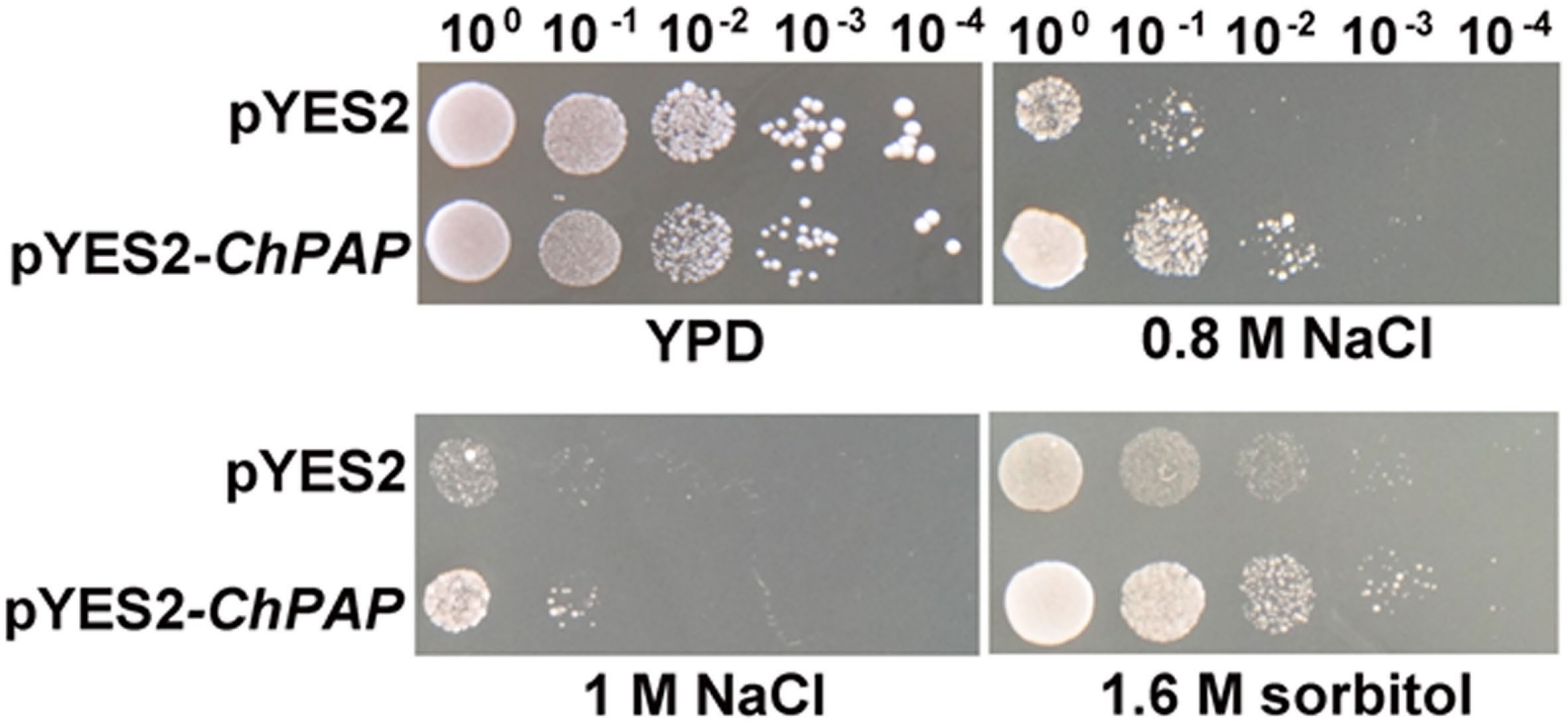
*ChPAP2*-overexpressing yeast cells exposed to NaCl and sorbitol stresses. Serial dilutions of yeast cells containing the pYES2 empty vector or pYES2-*ChPAP2* were independently spotted into solid yeast (1% yeast extract, 2% peptone, and 2% galactose) medium containing plates supplemented independently with 0.8-M NaCl, 1.0-M NaCl, and 1.6-M sorbitol.

The five serial dilutions of transgenic yeast cells were spotted into a solid yeast medium. The growth of yeast cells harboring the empty pYES2 vector was similar to that of the *ChPAP2* transgenic yeast in medium containing 1% yeast extract, 2% peptone, and 2% glucose without any stress. The *ChPAP2* transgenic yeast grew better than the controls in the presence of 0.8-M NaCl, 1-M NaCl, or 1.6-M sorbitol (Figure 3). Thus, the expression of *ChPAP2* in yeast cells improved the tolerance to NaCl and sorbitol, which indicated that ChPAP2 functions as lipid signaling molecule during abiotic stress (Munnik et al., 1996). *PAH1* encoded PAP1 was regulated to express under Zn deficiency and enhanced the activity of PAP enzyme (Soto-Cardalda et al., 2012). In addition, Arabidopsis PAP2 was found that was involved with ABA signaling and regulating the stomatal movements (Paradis et al., 2011). However, there was no relevant report on the investigation of PAP2 under abiotic stress.

### The Expression of *ChPAP2* Was Inducible Under NaCl and Sorbitol Stresses

**Figure 4:**
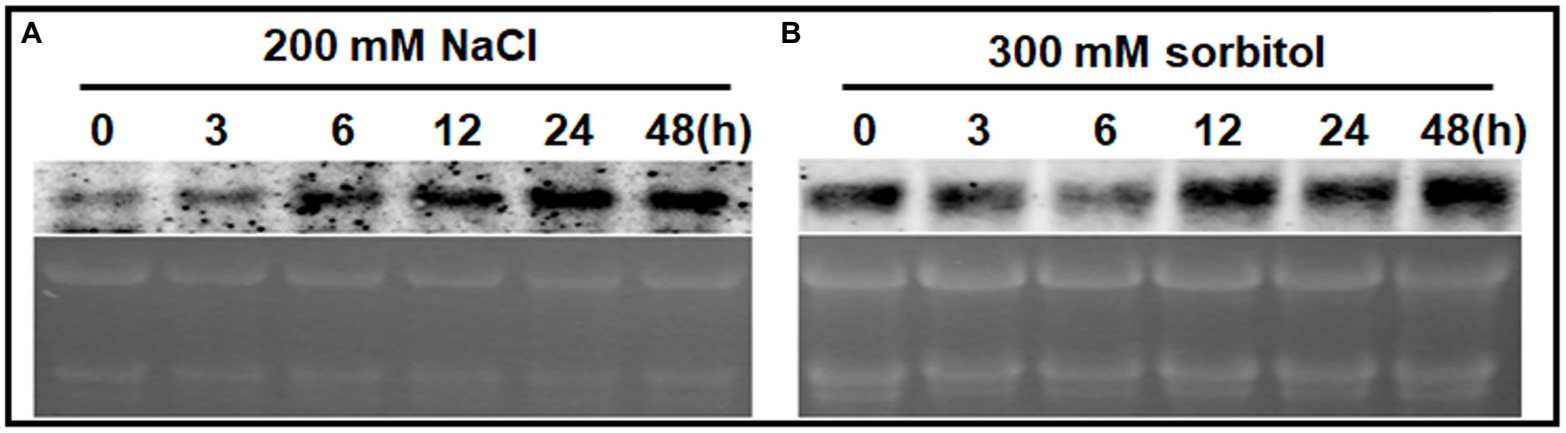
The mRNA expression levels of *ChPAP2* at various time points after exposure to NaCl- and sorbitol-stress treatments. Northern blot analyses of the *ChPAP2* gene’s expression levels in TSA *Chlorella* cells using a digoxigenin-labeled *ChPAP2* cDNA probe. The total RNA (5μg) was extracted from *Chlorella* cells treated independently with 200-mm NaCl **(A)** and 300-mm sorbitol **(B)**.

A northern blot analysis was used to detect *ChPAP2* expression patterns. Total RNA was used to analyze the effects of high salinity and drought stresses on *ChPAP2* expression. The TSA *Chlorella* cells were exposed independently to NaCl and sorbitol for 0, 3, 6, 12, 24, and 48h. The *ChPAP2* mRNA expression dramatically increased with the 200-mm NaCl treatment from 6 to 48h compared with the control (0h), indicating that *ChPAP2* was upregulated (Figure 4A). Thus, in yeast, the increased *ChPAP2* expression level may increase NaCl resistance. The PA and DGPP levels also increase under hyperosmotic stress conditions (Munnik et al., 2000). The presence of NaCl in liquid media not only leads to high salinity stress, but also to hyperosmotic stress in plants. We hypothesized that the *ChPAP2* expression level may be upregulated, allowing it to catalyze the excess DGPP into PA.

In this study, a sorbitol solution was used as the drought agent (Bocchini et al., 2018; Lu et al., 2019). The *ChPAP2* expression levels did not differ significantly compared with those of the control after 3–6h of exposure to 300-mm sorbitol. The *ChPAP2* mRNA level began to rise at 12h, and the level was the most significantly different from that of the control at 48h after treatment (Figure 4B). In *Saccharomyces cerevisiae*, PAP accumulates during exposure to hyperosmotic and dehydration stresses (Munnik et al., 2000). Thus, the upregulated *ChPAP2* expression may enhance drought tolerance.

### ChPAP2 Localized at the Plasma Membrane and in the Lumen of Vacuoles

**Figure 5:**
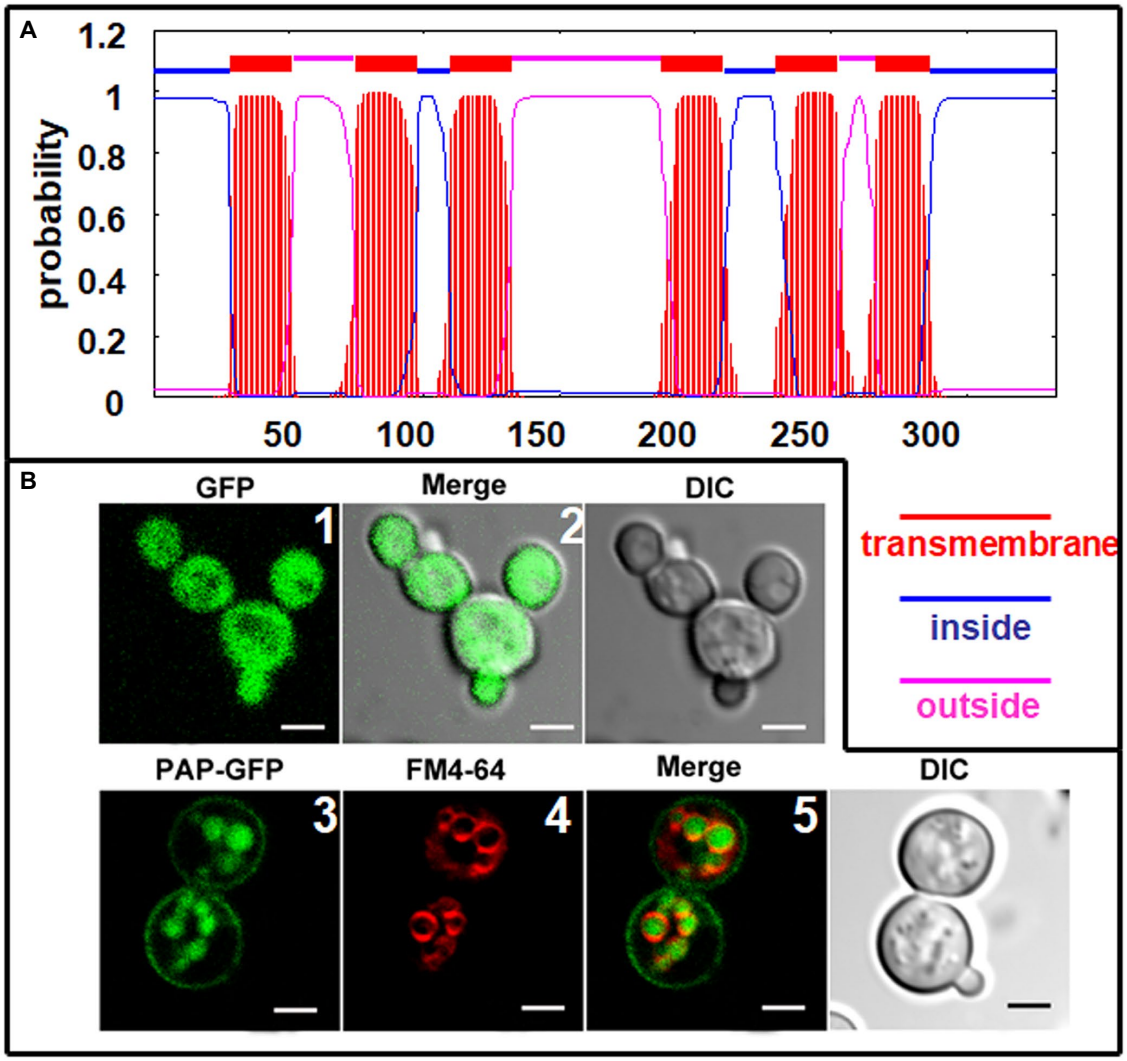
Subcellular localization of ChPAP2 in yeast cells. **(A)** Transmembrane helices in ChPAP2 (GenBank no. KT750011) were predicted using the TMHMM server v. 2.0; **(B)** the green fluorescence was observed using a confocal microscope. Upper: GFP gene expressed in the pYES2 vector and in the yeast strain INVSc1. Left, middle, and right panels show fluorescence, merged, and bright-field (DIC) images. Lower: *ChPAP2*-EGFP fusion gene expressed in the pYES2 vector and in the yeast strain INVSc1. Live cells overexpressing *ChPAP2*-EGFP were incubated with FM4-64. Left, second, third, and right panels show fluorescence, FM4-64, merged, and bright-field (DIC) images. Bars=5μm.

The deduced ChPAP2 amino acid sequence was predicted to contain six transmembrane domains (Figure 5A). To determine its subcellular localization, GFP was fused to the C-terminus of ChPAP2 (ChPAP2-GFP). The green fluorescence of the GFP protein alone was almost evenly distributed throughout the yeast cells (Figures 5B1,2). FM4-64 stained the vacuole membrane, and its localization signal was consistent with the vacuolar membrane signal (red fluorescence area; Figure 5B4). Thus, the ChPAP2 protein appeared to localize on the plasma membrane and in the lumen of vacuoles in yeast cells (Figures 5B3,5). The results suggested that ChPAP2 might play important roles in transporting lipids through the plasma membrane and in catalyzing DGPP into PA in the vacuoles.

ChPAP2, as a catalytic enzyme, participates in lipid metabolism. ChPAP2 localized on the plasma membrane may function in maintaining cell membrane stability and the ion balance of the cytoplasm in response to abiotic stresses. The ChPAP2 protein also is localized in the lumen of vacuoles, where it may be involved with lipid translocation and DGPP catalysis to form PA. Therefore, we hypothesized that ChPAP2 participates in catalyzing DGPP in the lumen of vacuoles.

The PAP protein may function as a signaling molecule in planta under stress conditions, and its levels accumulate during hyperosmotic and dehydration stresses (Munnik et al., 1996). Therefore, the PAP enzyme may play a role in regulating specific cellular DGPP and PA pools under stress conditions (Oshiro et al., 2003).

In summary, the *ChPAP* in TSA *Chlorella* was upregulated expression in treated with high salinity and drought, and the *ChPAP* in yeasts could tolerate high salinity and drought stresses. Its protein was localized at the plasma membrane and in the lumen of vacuoles. The ChPAP might translocate excess NaCl and sorbitol from plasma membrane and then segregate them into vacuole to regulate ion balance in the cytoplasm. As a consequence, the PAP as a novel transporter can enhance high salinity tolerance and accumulate excess high salinity. These characteristics make ChPAP for the bioremediation of saline-alkali soil.

## Data Availability Statement

The datasets presented in this study can be found in online repositories. The names of the repository/repositories and accession number(s) can be found in the article/supplementary material.

## Author Contributions

JW designed the research. QS, YR, and DS performed the experiments. HZ, JZ, and SG analyzed the data. KQ and AZ wrote the manuscript. All authors revised and approved the manuscript.

## Funding

This study was supported by the National Natural Science Foundation of China (grant nos. 31800200, 31902052, and 31972450), the Natural Science Foundation of Heilongjiang Province of China (grant no. YQ2020C002), and the Postdoctoral Research Initiation Funding Project of Heilongjiang Province (grant no. LBH-Q19084).

## Conflict of Interest

The authors declare that the research was conducted in the absence of any commercial or financial relationships that could be construed as a potential conflict of interest.

## Publisher’s Note

All claims expressed in this article are solely those of the authors and do not necessarily represent those of their affiliated organizations, or those of the publisher, the editors and the reviewers. Any product that may be evaluated in this article, or claim that may be made by its manufacturer, is not guaranteed or endorsed by the publisher.

## Footnotes

^1^ http://www.ncbi.nlm.nih.gov

^2^ http://www.cbs.dtu.dk/services/TMHMM/
